# Parallel and serial computing tools for testing single-locus and epistatic SNP effects of quantitative traits in genome-wide association studies

**DOI:** 10.1186/1471-2105-9-315

**Published:** 2008-07-21

**Authors:** Li Ma, H Birali Runesha, Daniel Dvorkin, John R Garbe, Yang Da

**Affiliations:** 1 Department of Animal Science, University of Minnesota, USA; 2 Supercomputer Institute, University of Minnesota, USA

## Abstract

**Background:**

Genome-wide association studies (GWAS) using single nucleotide polymorphism (SNP) markers provide opportunities to detect epistatic SNPs associated with quantitative traits and to detect the exact mode of an epistasis effect. Computational difficulty is the main bottleneck for epistasis testing in large scale GWAS.

**Results:**

The EPISNPmpi and EPISNP computer programs were developed for testing single-locus and epistatic SNP effects on quantitative traits in GWAS, including tests of three single-locus effects for each SNP (SNP genotypic effect, additive and dominance effects) and five epistasis effects for each pair of SNPs (two-locus interaction, additive × additive, additive × dominance, dominance × additive, and dominance × dominance) based on the extended Kempthorne model. EPISNPmpi is the parallel computing program for epistasis testing in large scale GWAS and achieved excellent scalability for large scale analysis and portability for various parallel computing platforms. EPISNP is the serial computing program based on the EPISNPmpi code for epistasis testing in small scale GWAS using commonly available operating systems and computer hardware. Three serial computing utility programs were developed for graphical viewing of test results and epistasis networks, and for estimating CPU time and disk space requirements.

**Conclusion:**

The EPISNPmpi parallel computing program provides an effective computing tool for epistasis testing in large scale GWAS, and the epiSNP serial computing programs are convenient tools for epistasis analysis in small scale GWAS using commonly available computer hardware.

## Background

The large number of SNPs available provides opportunities for detecting DNA variations associated with complex traits through GWAS using SNP markers. This is because an increased number of SNPs increases the chance that some SNPs may be DNA variations affecting phenotypes (direct SNP effects) or results in increased linkage disequilibrium (LD) with DNA variations that have direct effects on the phenotypes (indirect SNP effects). With high throughput SNP genotyping technology, SNP genotyping of a large number of individuals is becoming increasingly practical. Such large scale SNP genotyping increases the effectiveness of SNP association studies and provides an unprecedented opportunity to study complex genetic effects such as epistasis. The significance of epistasis (gene interaction) in complex or quantitative traits has been well recognized [1-5]. Large epistasis networks showing complex interactions among genes have been reported [6-8]. Fisher's partition [9] of the nine genotypic values of two bi-allelic loci into single gene effects (additive and dominance effects) and an epistasis effect assuming Hardy-Weinberg equilibrium (HWE) and linkage equilibrium (LE) laid the foundation of a quantitative genetics approach to study epistasis. Also assuming HWE and LE, Cockerham [10] and Kempthorne [11] partitioned Fisher's epistasis effect into four components using two different methods: additive × additive (A × A), additive × dominance (A × D), dominance × additive (D × A), and dominance × dominance (D × D) epistasis effects with the genetic interpretation of allele × allele, allele × genotype, genotype × allele, and genotype × genotype interactions respectively. This partitioning can be used as a tool for identifying the exact mode of a gene interaction effect. Kempthorne's partitioning of genotypic values has been extended to allow Hardy-Weinberg disequilibrium (HWD) and linkage disequilibrium (LD) so that Kempthorne's method could be used to test single-locus and epistasis effects in populations where HWD and LD may exist [12]. With genome-wide detection of epistasis effects, epistasis networks affecting a quantitative trait could be established. Computational difficulty is the main bottleneck for epistasis testing in large scale GWAS due to the large number of SNP combinations. The number of SNP combinations (M) is M = N(N - 1)/2 for testing two SNPs at a time, and is M = N(N - 1)(N - 2)/6 for testing three SNPs at a time, where N = number of SNPs. The computational difficulty of epistasis testing in large scale GWAS can be an open scale computing challenge that could exhaust the capabilities of any supercomputer in existence today. For example, pairwise epistasis testing of 1,000,000 SNPs would require 5 years using our EPISNP program and a single processor of the 2.66 GHz SGI Altix XE 1300 Linux cluster system at the Minnesota Supercomputer Institute, and this computing time could increase to 1.5 million years by adding just one SNP to the pairwise analysis (Table 1). With parallel computing, pairwise epistasis testing for any large scale GWAS currently in existence is possible. Large scale three-SNP epistasis testing may not be computationally feasible at this time. The parallel and serial computing software developed in this research is intended to provide computational tools for pairwise epistasis testing in GWAS on various parallel and serial computing platforms with the capability of pairwise epistasis testing for any large GWAS currently in existence.

**Table 1:** Estimated single-processor computing time on the SGI Altix XE 1300 Linux cluster system with 2.66 GHz Intel Clovertown processor, and the total number of tests for two-locus and three-locus analysis.

Number of SNPs (N)		Two-locus analysis	Three-locus analysis
500,000	Computing time (T)	T ≈ 1.2 years	T ≈ 200,000 years
	Number of tests (M)	M = (1.25) 10^11^	M = (2.08) 10^16^
1,000,000	Computing time (T)	T ≈ 5 years	T ≈ 1.5 million years
	Number of tests (M)	M = (5.0) 10^11^	M = (1.67) 10^17^

## Methods

The statistical methods implemented by the parallel and serial computing tools for detecting single-locus and epistasis effects include a general linear model for testing the marker effects of each SNP and each SNP pair, and include the extended Kempthorne model for testing additive and dominance effects of each SNP and for testing A × A, A × D, D × A, and D × D epistasis effects of each SNP pair. A two-step least squares analysis [13] is used to implement the statistical tests. The first step corrects the phenotypic values for systematic effects such as gender and age. This step estimates fixed non-genetic effects and then removes the estimated fixed non-genetic effects from the original phenotypic observations to obtain the corrected phenotypic values (or residual values). The second step conducts epistasis and single-locus tests using the corrected phenotypic values as the phenotypic observations. This two-step analysis estimates and removes systematic effects only once and hence has considerable computational advantage when the number of SNPs is large. The single-locus analysis tests three genetic effects: the SNP genotypic effect, additive effect, and dominance effect. The statistical model for testing single-locus effects is y = μ + SNP + e, where y = corrected phenotypic value, μ = common mean, SNP = the single-locus SNP genotypic effect, and e = random residual. The single-locus SNP genotypic effect was partitioned into additive and dominance effects. The single-locus genotypic effect answers the question whether the SNP had an effect on the phenotype whereas additive or dominance effect identifies the mode of the SNP effect. The statistical model for testing epistasis effects is y = μ + SNP1 + SNP2 + SNP1*SNP2 + e, where SNP1 and SNP2 are the two single-locus genotypic effects, and SNP1*SNP2 is the two-locus interaction effect (I-effect). The two-locus interaction effect was partitioned into four individual epistasis effects using the extended Kempthorne model that allows HWD and LD: A × A, A × D, D × A, and D × D epistasis effects. The two-locus interaction effect answers the question whether the two SNPs had an interaction effect whereas an individual epistasis effect (A × A, A × D, D × A, or D × D) identifies the mode of the interaction. The significance tests of the single-locus SNP effect and the two-locus interaction effect used an F-test. A t-test was used to test the significance of additive, dominance and epistasis effect using the following formula

$${\hat{T}}_{x}=\frac{L_{x}}{\sqrt{\hat{\mathrm{var}}(L_{x})}}=\frac{s_{i}\hat{g}}{\sqrt{s_{i}{\left(X^{\prime }X\right)}^{-1}{s^{\prime }}_{i}s^{2}}}$$

where *L*_*x *_= contrast to estimate the genetic effect, *s*^2 ^= (**y **- **Xĝ**)*' *(**y **- **Xĝ**) (*n *- *k*) = estimated residual variance, **ĝ **= the least squares estimates of the SNP genotypic effects, and **s**_i _= a function of marginal and conditional allelic and genotypic frequencies for estimating genetic effect i, which is either additive, dominance or an epistasis effect, and where n = number of observations and k = rank of **X **[12]. For testing epistasis effects involving the X chromosome in mammals (or Z chromosome in birds), only females (or males in birds) can be included in the analysis. For epistasis analysis involving SNPs in pseudoautosomal regions, the analysis is the same as for autosomal SNPs. These epistasis testing methods were implemented in a parallel computing program intended for larges scale GWAS and in a serial computing program intended for small scale GWAS that could be analyzed on commonly available computer hardware.

Minimizing the processor memory required is critical to developing an efficient and successful parallel computing program because each individual processor has a limited amount of memory available. For example, each core of the quad-core processor on the SGI Altix XE 1300 Linux cluster system with 2.66 GHz Intel Clovertown processors (Calhoun) at the Minnesota Supercomputer Institute has a limit of 2 GB of memory. Therefore, the parallel code should use as little processor memory as possible to achieve scalability for large scale analysis that will otherwise require large processor memory. A two dimensional SNP data distribution scheme (Table 2) among processor cores was designed to minimize the memory requirement of each processor. To assign SNPs to each processor core, the N SNPs are evenly divided into m subsets with n SNPs in each subset such that the total number of processor cores (p) to be used is p = m(m+1)/2. For simplicity, n = N/m is assumed to be an integer. In the case N/m is not an integer, the leftover SNPs are assigned to an extra core. In Table 2, each diagonal processor core receives one subset of n SNPs and computes [3n + 5n(n - 1)/2] tests, and each off-diagonal processor core receives two subsets of SNPs (2n SNPs) and computes 5n^2 ^pairwise tests. Therefore, only (2n)/(mn) = 2/m of the N SNPs are stored in each off-diagonal processor core, and only 1/m of the N SNPs are stored in each diagonal processor core defined in Table 2. As the number of processor cores (p) increases, the number of SNP subsets (m) increases and the memory required for each processor core decreases. Therefore, the increased memory requirement per processor core for large scale SNP analysis can be reduced by increasing the number of processor cores used. The parallel computing code was optimized to minimize inter-processor communications and was crafted for portability to various parallel and serial computing platforms. Testing results showed that the parallel computing code achieved excellent speedup and scalability and achieved excellent portability, as to be discussed below.

**Table 2:** Example of distributing N SNPs to m(m+1)/2 processor cores (P_i_, i = 1, m(m+1)/2) for the case where N/m is an integer, where m = N/n = number of subsets of SNPs with each subset having n SNPs (m and n are assumed integers).

Subset 1: SNP_1 _... SNP_n_	Subset 2: SNP_n+1_... SNP_2n_	... ...	Subset m: SNP_n(m-1)+1 _... SNP_N_	
	
P_1_	P_2_	... ...	P_m_	Subset 1: SNP_1 _... SNP_n_
		
	P_m+1_	... ...	P_2m-1_	Subset 2: SNP_n+1_... SNP_2n_
		
		... ...	... ...	... ...
		
			P_m(m+1)/2_	Subset m: SNP_n(m-1)+1 _... SNP_N_

Each diagonal core receives one subset of n SNPs and computes [3n + 5n(n - 1)/2] tests, and each off-diagonal core receives two subsets of total 2n SNPs and computes 5n^2 ^tests.

## Results

A parallel computing program named EPISNPmpi and a serial computing program named EPISNP were developed for genome-wide pairwise epistasis testing. Three serial computing utility programs were developed to estimate computing time, to produce graphical chromosome view of significant single-locus results, and to produce graphical display of epistasis network.

### The EPISNPmpi and EPISNP programs

The EPISNPmpi and EPISNP programs provide two sets of SNP tests: single-locus analysis and pairwise analysis. The single-locus analysis tests three effects of each SNP: SNP genotypic effect (M), additive (A) and dominance (D) effects. The pairwise analysis tests five effects of each pair of SNPs: The I-effect, A × A, A × D, D × A, and D × D. Three input files in text format are required, the phenotype file, the SNP genotype file, and the parameter file. The phenotype file contains observations of the quantitative trait(s), family ID, individual ID, individual gender, and non-genetic fixed effects such as smoking status and age of each individual. The SNP genotype file contains family ID, individual ID, individual gender, and SNP genotypes, and should be one file for each chromosome. The parameter file with file name parameter.dat provides various user-specified controls for the EPISNPmpi and EPISNP programs to have the flexibility to be generally applicable. These controls include the number of quantitative traits to be analyzed, user specified number of chromosomes, code for the sex chromosome, formats for SNP genotypes and missing values, and user specified number of fixed non-genetic factors to be included in the statistical model, where a fixed non-genetic factor can be an indicator variable or continuous variable (covariable). Both EPISNPmpi and EPISNP programs are applicable to populations with HWD and LD.

**Figure 1:**
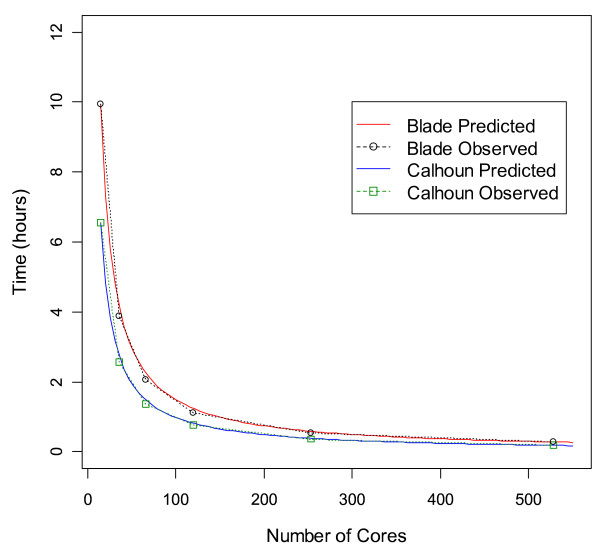
**Observed and predicted run times of the EPISNPmpi program on Minnesota Supercomputing Institute's 2.6 GHz IBM BladeCenter Linux cluster (Blade) and the SGI Altix XE 1300 Linux cluster system with 2.66 GHz Intel Clovertown processor (Calhoun)**. The observed run times (circles representing Blade and squares representing Calhoun) matched well with the predicted run times under ideal speedup and scalability (solid line representing Blade and dotted line representing Calhoun). Analyses in this figure used a hypothetical GWAS data set with 50,000 SNPs and 2000 individuals.

The speedup and scalability [14,15] of the EPISNPmpi parallel program were evaluated for two supercomputer systems: a 2.6 GHz AMD Opteron IBM BladeCenter Linux cluster (Blade) and the Calhoun system. In parallel computing, speedup refers to how much a parallel algorithm is faster than a corresponding sequential algorithm and is defined as S_k _= T_1_/T_k_, where k = number of processors, T_1 _= the execution time of the sequential algorithm with one processor-core, and T_k _= the execution time of the parallel algorithm with k processor-cores. Linear or ideal speedup is achieved when S_k _= k. Scalability refers to the stability of average performance of a parallel program as the number of processors increases. Ideal scalability is achieved when the efficiency of k processors (E_k_) is E_k _= S_k_/k = 1. Figure 1 shows the observed and predicted computing time using 15–528 processor cores, where each processor consists of four cores. The predicted computing time was calculated using the following formula assuming an ideal speedup or scalability

(1) t_k _= t_1_/k

where k = number of processor cores, t_k _= computing time using k processor cores, and t_1 _= computing time using one processor core. In Figure 1, the computing times were normalized to the computing time on 15 processor-cores because the minimal number of cores used was 15. Results in Figure 1 showed that the observed computing time and the predicted computing time assuming ideal speedup and scalability matched very well, indicating that the EPISNPmpi coding achieved excellent speedup and scalability. Based on the observed run times of 0.20 and 19.3 hours for 50,000 and 500,000 SNPs respectively using 528 cores of the Calhoun system, the estimated computing time for pairwise epistasis tests is approximately an increasing quadratic function of the number of SNPs. Let N = the number of SNPs and N_0 _= a smaller number of SNPs with a known computing time (t_0_) for running EPISNPmpi such that N = N_0 _(x). Then, the computing time required for analyzing N SNPs (t_N_) is approximately

(2) t_N _= (t_0_)(x^2^)

The run time of 19.3 hours for 500,000 SNPs using 528 cores showed that pairwise epistasis testing for GWAS with about 500,000 SNPs could be completed in one day using about 25% of the 2048 cores of the Calhoun system. Based on this computing time and equations (1–2), the predicted time for pairwise epistasis testing among 1,000,000 SNPs using all 2048 cores of the Calhoun system would require about 20 hours to complete. This prediction indicates that EPISNPmpi is capable of completing pairwise epistasis analysis in one day for any large scale GWAS currently in existence, noting that the numbers of SNPs used in current large scale GWAS are in the range of 500,000 ~ 940,000, as represented by NIH's GAIN projects [16]. Sample size, or the number of individuals, affects the computing time as well, but the increase in computing time due to increased sample size is minor. The EPISNPmpi code is highly portable to various computing platforms and has been ported to all supercomputer systems at the Minnesota Supercomputer Institute and to several popular serial computing platforms.

The EPISNP program is designed for epistasis analysis in small-scale GWAS on commonly available computer hardware. For example, an analysis of 5700 SNP markers took about 18 hours to complete on a PC with a single 3.8 GHz Pentium 4 processor.

The EPISNPmpi and EPISNP programs each produces two output files of the most significant results of single-locus tests and two output files of the most significant results of pairwise epistasis tests. The output file for significant epistasis results currently displays the names and chromosome locations of the two SNPs in each SNP pair with significant I-effect (interaction between the two loci), A × A, A × D, D × A, or D × D effect, significance level (p-value), and ordered estimates of individual effects that are useful for identifying the best and worst gene combinations affecting a phenotype [17]. The second output file of single-locus tests is used as the input file of the EPISNPPLOT program and the second output file of pairwise epistasis tests is used as the input file of the EPINET program.

### Three serial computing utility programs

**Figure 2:**
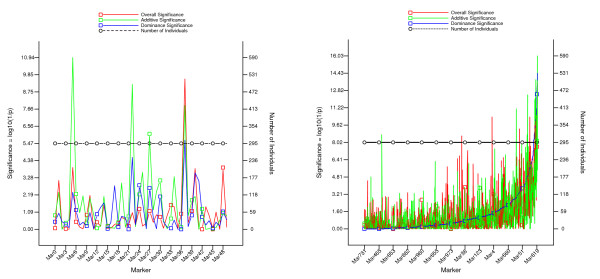
**Examples of chromosome view of single-locus significance and sample size produced by the EPISNPPLOT program that draws chromosome views for all chromosomes by one command**. The figure on the left is an example of chromosome view based on the original marker order, and the figure on the right is an example of chromosome view in ascending order of significant dominance effects.

**Figure 3:**
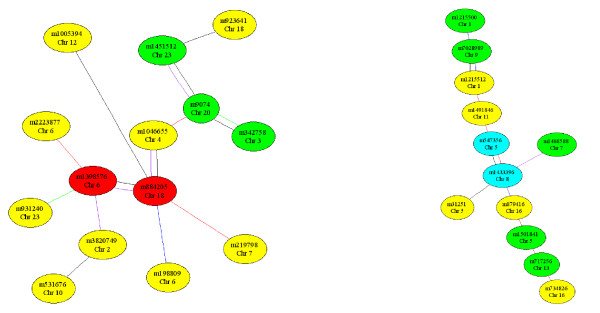
**Examples of SNP epistasis network of a phenotype produced by the EPINET program that by default draws the 10 largest epistasis networks from the input test results**. Line color: black = I-effect, red = A × A, purple = A × D, blue = D × A, green = D × D. Node color: red: p < 10^-8^, cyan: p < 10^-7^, green: p < 10^-6^, yellow: p < 10^-5^.

The EPISNPPLOT program plots the chromosome view figures, where each figure shows the significance of each of the three single SNP effects and the sample size for all SNPs on each chromosome (Figure 2). The program produces chromosome view figures for all chromosomes by one command using an output file from EPISNPmpi or EPISNP as the input file. These chromosome views help identify chromosome regions with various degrees of significant effects and markers that did not have sufficient information to yield significant effects. By default, the EPISNPPLOT program draws chromosome view figures in the original marker order as in the input file. The user has the option to sort the input data by the marker significance, additive significance, dominance significance, or the number of observations. In Figure 2, the figure on the left is an example of a chromosome view based on the original marker order, and the figure on the right is an example of a chromosome view in ascending order of significant dominance effects. The EPINET program draws figures of epistasis networks of SNPs with significant epistasis effects at four user specified p-values. The program requires two input files: the parameter file to specify four significance levels (p values) for selecting loci in the epistasis networks, and the effect file that contains epistasis testing results from EPISNPmpi or EPISNP. The default input is to use 'effects.dat' as the input file and to print the 10 largest networks (Figure 3). Alternatively, the user can specify the file name on the command line. If the input file is specified, the number of networks to print can also be specified. The EPINET program uses four user specified node colors to represent the four significance levels defined by the corresponding p-values, and five program defined line colors to denote the five types of epistasis effects (Figure 3). The CPUHD estimates CPU time required to complete the data analysis using the EPISNP program and the total storage space required to store the output files. This is helpful for planning an epistasis analysis. For example, a potentially excessively long running time can be avoided by running CPUHD first. Detailed instructions for using EPISNPmpi, EPISNP and the three utility programs described below are available in two user manuals [17,18].

### Commodity cluster-based processing of EPISNPmpi

EPISNPmpi has been developed and tested on many modern high-performance computers and supercomputer systems. Price-to-performance ratio of the computing system can be an important consideration in practice. To utilize commonly available computer hardware for high performance computing, EPISNPmpi has been implemented to run on commodity cluster or on an inexpensive network of workstations using MPICH message passing libraries. MPICH is a portable implementation of MPI, a standard for message-passing for distributed-memory applications, and is freely available at .

## Discussions

Computational difficulty is the main bottleneck of epistasis testing in large scale GWAS. The computing tools we have developed help address the computational difficulty in epistasis analysis in large scale GWAS. The computing speed can be further improved if a more powerful computer system is used. However, serious computational challenges still exist in at least three areas: 1) Increased number of SNPs used in GWAS, 2) Integration of GWAS and a gene expression study, and 3) Joint epistasis testing for three or more SNPs at a time. The human genome has about 10 million SNPs. Although an exhaustive analysis of all human SNPs is not yet a reality, the number of SNPs used in GWAS is clearly rapidly increasing. Since the computing time for epistasis testing increases approximately as a quadratic function of the number of SNPs, computing difficulty will rapidly increase as the number of SNPs increases. Integration of large scale GWAS and a gene expression study using the same individuals poses another serious computational challenge. In this case, the computing time required is multiplied by the number of genes, where gene expression intensity of each gene is treated as one phenotype [19]. The joint epistasis testing for three or more SNPs could be the ultimate computing challenge. As shown in Table 1, adding just one SNP to the pairwise epistasis test for 1,000,000 SNPs could require 1/3 million times as much computing time. A tempting solution would be to test epistasis effects for a subset of SNPs with significant single-locus effects. However, this is not a good idea because requiring significant main effects for epistasis testing could miss many or even all significant epistasis effects with stringent p-values to declare significance. For example, the significant epistasis effects with p < 10^-7 ^for 5700 SNPs covering all 23 human chromosomes reported in Ma et al. [20] did not involve any SNPs with significant single-locus at p < 10^-4^. Therefore, requiring significant single-locus effects at p < 10^-4 ^would have missed all the ten significant epistasis effects at p < 10^-7 ^among the 5700 SNPs. The EPISNPmpi and EPISNP programs provide capabilities for testing all possible pairwise epistasis effects. However, the use of these programs should be considered as only one step in GWAS analysis. Considerable work still may be required for digesting the test results.

## Conclusion

The EPISNPmpi parallel computing program provides a computing tool capable of completing pairwise epistasis tests in large scale GWAS in a timely manner using a supercomputer system. The serial computing programs can be useful and convenient tools for epistasis analysis in small scale GWAS using commonly available computer hardware. EPISNPmpi is a portable program which not only exploits the capability of supercomputers but also runs on inexpensive loosely coupled cluster systems.

## Availability and requirements

**Project name: **Parallel and serial computing for genome-wide SNP analysis

**Project homepage: **

Operation systems:

1. EPISNPmpi :

EPISNPmpi is the parallel computing program for testing single-locus and pairwise epistasis effects and is available for running on the majority of parallel computer systems. The following are the currently supported processors type, MPI libraries, compilers and corresponding binaries (see Table 3):

**Table 3:** Currently supported processors type, MPI libraries, compilers and corresponding binaries

***MPI library***	***Compiler***	***Processor***	***Binary***
Voltaire MPI	Intel	Intel	EPISNPmpi_2.0_Voltaire_intel_intel.tar.gz
Voltaire MPI	Intel	AMD	EPISNPmpi_2.0_Voltaire_intel_AMD.tar.gz
Voltaire MPI	Intel	Intel (EM64T)	EPISNPmpi_2.0_Voltaire_suse_EM64T.tar.gz
PathMPI	Pathscale	AMD	EPISNPmpi_2.0_Pathscale_suse_AMD.tar.gz
IntelMPI	Intel	AMD	EPISNPmpi_2.0_intelMPI.suse_AMD.tar.gz
OpenMPI	Intel	Intel (EM64T)	EPISNPmpi_2.0_OpenMPI_suse_EM64T.tar.gz
IBM MPI	Intel	Power4	EPISNPmpi_2.0_IBM_AIX_pwr.tar.gz
MPT	Intel	Itanium	EPISNPmpi_2.0_SGI-Altix_SUSE_itanium.tar.gz

2. epiSNP :

The epiSNP package consists of four serial computing programs, EPISNP, CPUHD, EPISNPPLOT, and EPINET. EPISNP is the serial computing program for testing single-locus and pairwise epistasis effects. The following are the currently supported operation systems, processors types, and compilers used to generate binaries (see Table 4):

**Table 4:** Currently supported operation systems, processors types, and compilers used to generate binaries

***Operation system***	***Compiler***	***Processor***	***Binary***
Widows	Intel	Intel/AMD	epiSNP_2.0_Widows.zip
Irix	SGI	MIPS	epiSNP_2.0_SGI_Irix_Mips.tar.gz
Linux (SUSE)	Intel	AMD	epiSNP_2.0_intel_suse_AMD.tar.gz
Linux (SUSE)	Intel	Intel (EM64T)	epiSNP_2.0_intel_suse_EM64T.tar.gz
Linux	Portland	Intel (32bit)	epiSNP_2.0_Linux_Portland_Intel.tar.gz
Linux (SUSE)	Pathscale	AMD	epiSNP_2.0_Pathscale_suse_AMD.tar.gz
Unix (AIX)	XLF	Power4	epiSNP_2.0_xlf_AIX_power.tar.gz

In the above binaries, epiSNP_2.0_Windows.zip contains all the four programs (EPINET, CPUHD, EPISNPPLOT, EPINET), while each of the other .gz file contains EPISNP and CPUHD only.

**Other requirements: **None.

**License: **None.

**Any restrictions to use by non-academics: **None.

## Abbreviations

GWAS: genome-wide association study; SNP: single nucleotide polymorphism; LE: linkage equilibrium; LD: linkage disequilibrium; HWE: Hardy-Weinberg equilibrium; HWD: Hardy-Weinberg disequilibrium; I-effect: two-locus interaction effect; A × A: additive × additive epistasis effect; A × D: additive × dominance epistasis effect; D × A: dominance × additive epistasis effect; D × D: dominance × dominance epistasis effect; Blade: 2.6 GHz IBM BladeCenter Linux cluster at the Minnesota Supercomputer Institute; Calhoun: the SGI Altix XE 1300 Linux cluster system with 2.66 GHz Intel Clovertown processor at the Minnesota Supercomputer Institute.

## Authors' contributions

LM is the main author of EPISNPmpi and is the author of EPISNP and CPUHD programs. HBR directed the development of the parallel computing coding and directed the work of porting the parallel computing code to various parallel and serial computing platforms including commodity cluster processing, and did a portion of the coding of the EPISNPmpi program. DD is the author of the EPISNPPLOT program. JRG is the author of the EPINET program. YD coordinated this research, designed most functions of the computing tools, and is the lead writer of the manuscript. All authors read and approved this manuscript.
